# Biological identity of orbital cavernous venous malformations

**DOI:** 10.5935/0004-2749.2023-0338

**Published:** 2024-04-03

**Authors:** Ana Carolina Lourenço Veloni Longhim, Fernando Chahud

**Affiliations:** 1 Department of Pathology and Forensic Medicine, Faculdade de Medicina de Ribeirão Preto, Universidade de São Paulo, Ribeirão Preto, SP, Brazil

**Keywords:** Orbital neoplasms, Vascular malformations, Vascular system injuries, Cavernous hemangioma, Cavernous venous malformations, Biomakers, tumor, Endothelium, vascular, Cell proliferation

## Abstract

Vascular anomalies comprise a wide spectrum of clinical manifestations related to disturbances in the blood or lymph vessels. They correspond to mainly tumors (especially hemangiomas), characterized by high mitotic activity and proliferation of the vascular endothelium, and malformations, endowed with normal mitotic activity and no hypercellularity or changes in the rate of cell turnover. However, the classifications of these lesions go beyond this dichotomy and consist various systems adapted for and by different clinical subgroups. Thus, the classifications have not reached a consensus and have historically caused confusion regarding the nomenclatures and definitions. Cavernous venous malformations of the orbit, previously called cavernous hemangiomas, are the most common benign vascular orbital lesions in adults. Herein, we have compiled and discussed the various evidences, including clinical, radiological, morphological, and molecular evidence that indicate the non-neoplastic nature of these lesions.

## INTRODUCTION

### General aspects of vascular lesions

Vascular lesions comprise a wide spectrum of clinical manifestations related to morphological, structural, or functional alterations of vessels^(1-8)^, and their classification has been the subject of discussions and discrepancies for decades. In 1982, Mulliken et al. defined the basic differential characteristics between tumors (mainly hemangioma) and malformations, the two main types of vascular lesions observed in clinical practice. Hemangiomas demonstrate hypercellularity and high mitotic activity of the endothelial cells. However, malformations demonstrate normal mitotic activity, without hypercellularity or changes in the turnover rate^(9)^.

The term hemangioma is nonspecific and has been used to describe vascular anomalies of almost all etiologies, morphologies, and natural histories^(9-11)^. This has caused challenges when classifying and, more importantly, diagnosing diseases. The term “cavernous hemangioma” is especially controversial in two aspects. It has been used indiscriminately to describe several lesions located in different anatomical sites^(9,12-16)^, and it usually implies a benign neoplastic lesion. For more than a decade, several studies have demonstrated that most orbital lesions, if not all, are malformations^(14,17,18)^.

### Cavernous venous malformation of the orbit

Cavernous venous malformation (CVM), formerly known as “cavernous hemangioma,” is the most common benign orbital vascular anomaly in adults^(18,19)^, accounting for 9% of all reported cases^(20,21)^. Their characteristic morphological architecture includes large channels and vascular spaces filled with blood. Although CVMs have been described in children^(22)^, they usually remain asymptomatic until adulthood, manifesting around the 4^th^ or 5^th^ decade of life. CVMs are more prevalent in women than men, and some studies indicate a possible hormonal modulation in the clinical course of the disease^(23-25)^.

More than 80% of CVMs are located in the intraconal orbital compartment, and they commonly lie lateral to the optic nerve^(26)^. CVMs usually appear as a single and unilateral lesion, with a preference for the left orbit^(27)^. Bilateral or multiple lesions have been described, but they are quite rare^(28-30)^. They may occur in association with systemic diseases such as Blue rubber bleb nevus syndrome^(31,32)^ and Maffucci’s syndrome^(33)^.

The most common clinical sign of CVMs is progressive and painless axial proptosis, which affects approximately 70% of all patients^(34)^. CVMs can occasionally cause mild visual impairment, retinal streaks, papilledema, blurred vision, double vision, eyelid swelling, pain, mechanical ptosis, corneal exposure, and gaze-evoked amaurosis^(18,21,23,34-36)^. Acute symptoms such as hemorrhage and periorbital ecchymosis rarely occur^(37-39)^. Symptoms are usually reversible unless the injury causes a permanent axial length change or optic nerve dysfunction^(35)^. When a CVM is clinically suspected, imaging tests such as ultrasound, computed tomography (CT), or magnetic resonance imaging (MRI) are useful for making a definitive diagnosis^(40)^.

The management of an orbital CVM usually entails surgical resection, which produces satisfactory results because the lesion is completely encapsulated and does not adhere to the orbital contents^(41)^.

Despite the numerous studies that have been published on the clinical behavior of and different surgical approaches to these orbital lesions, literature on their pathogenesis and cellular and molecular characterization are limited. These data contribute to elucidating their biological status. Thus, herein, we aimed to present scientific evidence that indicates CVMs are non--neoplastic in nature.

## METHODS

We conduct a review of the main studies that have investigated orbital CVMs, with particular emphasis on clinical, radiological, and morphological evidences, including the immunophenotypic aspects that indicate that this lesion is an atypical malformation rather than a neoplasm. An advanced PubMed search was performed using the following keywords: “vascular lesions,” “vascular anomalies,” “vascular malformations,” “cavernous malformations,” and “cavernous hemangiomas” with and without the keyword “orbital.” Studies with an abstract and full text in Portuguese, English, French, Spanish, and German were screened. The most relevant studies addressing the nature of the injury were selected up to a maximum of 100 references.

## RESULTS

### Clinical evidence

True hemangiomas such as an infantile hemangioma (IH), a variant of capillary hemangiomas and the most common orbital tumor in children, typically appear during the first two months of life as an erythematous patch, telangiectasia surrounded by a whitish halo, or a depigmented area^(42)^. Subsequently, they demonstrate a proliferative phase with rapid expandable growth. Although benign, they can reach large dimensions, occasionally becoming locally destructive^(43)^. Thereafter IHs reach a growth plateau, followed by a phase of involution which is characterized by a decrease in their size^(10)^. The lesion can demonstrate an infiltrative capacity, mainly affecting the adipose and muscle tissue^(11,44)^. Additionally, IHs can arise in several areas simultaneously^(45)^.

Most patients with orbital CVMs develop symptoms late in life, approximately the 4^th^ or 5^th^ decade of life. Unlike IHs, CVMs are slow-growing lesions that may remain stable for years, even returning to a quiescent stage after a period of growth^(46)^. McNab demonstrated that orbital CVMs are often incidentally detected in asymptomatic patients. However, over time, a significant proportion of these lesions grow, produce symptoms, and eventually require treatment^(47)^. There is no report of observable infiltration of adjacent structures by CVMs or metastases, despite CVMs being multifocal^(48-51)^ and rarely incorporating local structures in their fibrous capsule^(34)^. Malignant transformation of these lesions has never been documented^(11)^.

### Radiological evidence

Orbital CVMs have a typical pattern on imaging that allows easy diagnosis, is reliable, and does not require a biopsy^(17)^. On computed tomography angiography, these lesions appear as a homogeneous noninfiltrative mass with regular borders and irregular and delayed focal enhancement on contrast administration^(52,53)^. On MRI, these lesions typically appear isointense on T1-weighted images and hyperintense on T2-weighted images. After administration of gadolinium contrast, they enhance dynamically, in a total and homogeneous presentation^(14,17)^. Typically, these lesions do not expand during the Valsalva maneuver, and this imaging pattern is quite characteristic of slow-flow venous malformations without an arteriovenous shunt or distensible components^(5)^. In clinical practice, these characteristics can help differentiate CVMs from true neoplasms such as schwannomas, hemangiopericytomas, or solitary fibrous tumors^(54)^.

### Histological evidence

On microscopic examination, IHs are solid proliferations of endothelial cells with multilaminated basement membranes and a fibrous septum delineating a lobular architecture; these findings are absent in normal tissues or in malformations^(10)^. IHs consist of anastomosed vascular channels with an infiltrative growth pattern, and a true capsule is absent^(55)^. In its proliferative phase, IH demonstrates a characteristic histopathological pattern with enlarged endothelial cell masses and frequent mitotic figures; some endothelial cells attempt to form vascular spaces with small and irregular lumens^(11,45)^. The neoplastic nature of these lesions is evident from the presence of these mitotic figures, and unlike normal endothelial cells, their derivatives readily grow in *in vitro* cultures^(56)^. At the beginning of the involution phase, mature cells become more abundant, the endothelium becomes less active and progressively flattens, and luminal and intervascular fibrosis increases. Electron microscopic examination demonstrates that endothelial cells appear to be surrounded by pericytes^(42)^.

Orbital CVMs are well-encapsulated lesions with complex networks of dilated and ectatic vascular channels that are lined by flat and normal endothelial cells. They demonstrate varying amounts of smooth muscle cells in their walls and are surrounded by moderately thick collagenous fibrous tissue^(11,23)^. They tend to demonstrate greater amounts of thrombosis, stromal expansion, and myofibroblastic proliferation than IHs, and do not demonstrate lymphatic elements^(5,57)^. No vessel demonstrates an internal elastic lamina. Thickened vessels, if present, are the result of thrombosis and recanalization, which demonstrates their venous nature^(5)^. On electron microscopy, the elastic lamina is absent in vascular channels, which are surrounded by multiple layers of well-differentiated, trichromium-positive, fusiform muscle cells; these characteristics are not seen in classical hemangiomas^(34,42)^.

Most of the morphological characteristics of CVMs were described in the study by Harris et al. in the 1970s, who noted a proliferation of endothelial cells around intralesional thrombi^(34)^. Additionally, Garner observed the presence of myofibroblasts, which was described as an apparently random distribution of smooth muscle blocks within fibrous trabeculae^(58)^. The use of modern staining techniques makes it possible to contextualize this histopathological evidence in terms of natural processes occurring in slow-flowing venous systems, mainly thrombosis and recanalization processes^(5,11)^.

## IMMUNOPHENOTYPING

### Endothelial immunophenotype

Among immunohistochemical markers, the CD31 antigen is considered the most specific for vascular lesions. CD34 demonstrates specificity for vascular and lymphatic endothelium, and the D2-40 antigen, also called podoplanin, is a relatively specific marker of the lymphatic endothelium^(11)^. Immunohistochemical studies have revealed that orbital CVMs and IHs demonstrate positive staining for markers, CD31 and CD34, and negative staining for podoplanin. Thus, neither lesion in their classic form demonstrates lymphatic components^(5,11,13,59-63)^.

Cavernous hemangioma cells from sites such as skin, mucosa, and liver demonstrate immunonegativity for Prox-1, a specific marker for the differentiation of lymphatic endothelial cells^(63)^. However, data on the expression of Prox-1 in orbital CVMs were not found in the literature at the time of conclusion of this review.

Glucose transporter type 1 (GLUT-1), which is widely expressed in fetal tissues, is a sensitive and highly active immunohistochemical marker specific for endothelial cells of IHs. GLUT-1 is observed both in the proliferative and involution phases of IHs^(64)^, demonstrating its strong vasculogenic potential^(65-68)^. Orbital CVMs and venous and lymphatic malformations are immunonegative for GLUT-1, making it an essential marker of the different natures of these lesions^(11)^.

### Smooth muscle cell immunophenotype

The smooth muscle actin (α-SMA) marker detects the presence of contractile myofilaments in the cells of vascular channel walls as well as in the myofibroblasts of the stroma. The staining pattern of SMA in CVMs demonstrates wide variation in the wall thickness of cavernous channels and several irregularities with focal outgrowths of desmin-negative mural cells^(11)^. Negative labeling for this marker implies incomplete smooth muscle differentiation in CVMs, which correlates with myofibroblasts. The disorganized nature of the muscle walls within the same luminal unit and the interruption of myofibroblastic differentiation are powerful data that support the hypothesis that the lesion is a malformation^(11)^.

### Presence of intraluminal thrombosis and its role in lesion growth

The presence of a large amount of intraluminal thrombosis is a classic histopathological evidence in orbital CVMs^(5,11,18)^. Immunophenotyping study results indicate that all the mechanisms involved in thrombus formation and its resolution, as well as in neovascularization, are simultaneously evident in different regions of the same lesion^(5,11,34)^. The presence of positive staining for CD31^(5,11,13,61-63)^ indicates the initial lining by endothelial cells in the thrombus and formation of neovascular networks^(5)^. Ki-67, a cell proliferation marker, is expressed in areas of periluminal hypercellularity, which may be evidence of the proliferative phase of thrombus formation^(69)^. Expression of VEGF and its receptor Flk-1 is upregulated, which results in neovascular demodulation and growth of the lesion^(62)^. Subsequently, stromal remodulation, reabsorption, and recanalization of the thrombus occurs, followed by proliferation of fibroblasts and myoid cells with positive staining for α-SMA and negative staining for desmin. Thereafter, the interstitial spaces harden and expand^(70)^.

### Pro-angiogenic regulators and lesion growth dynamics

The VEGF family of proteins plays a crucial role in vasculogenesis and lymphangiogenesis in both normal and pathological situations. Signaling is mediated mainly by VEGFr1/Flt-1 and VGFRr2/Flk-2, which are located on endothelial cells, as well as VEGFr3, which is involved in the regulation of lymphatic vessel development^(70)^. Histopathological studies have demonstrated that orbital CVMs express VEGF-A and its Flt-1 and Flk-1 receptors^(5,61,62,71)^, which may be associated with the neovascular demodulation caused by the thrombosis and recanalization processes in the lesions. Thus, VEGF, the main effector and promoter of endothelial proliferation and differentiation, may also be involved in the growth of CVMs^(70)^. The growth of CVMs, unlike in classical proliferative hemangiomas, is attributed to ectasia and hypertrophy, which in turn are attributable to hormonal changes or local hemodynamic disturbances resulting from slow blood flow, ischemia, or hypoxia^(60)^.

In 2013, Osaki et al. observed small outbreaks of CD31/34-positive endothelial cells in the stroma of analyzed CVM lesions with early lumenization in segments unaffected by thrombi. These findings support the hypothesis that the expression of growth factors may represent a form of slow and progressive growth, which is typical of orbital CVMs. bFGF is also expressed in these lesions, and they may stimulate the growth of endothelial cells and vascular smooth muscle cells, suggesting that this marker may participate in addition to VEGF in anomalous growths^(11)^. Therefore, the presence of thrombosis could explain why orbital CVMs suddenly expand through the proliferation of vascular channels and progressive ectasia without involvement of proliferative processes. This may be the starting point of the growth of these lesions^(47)^.

### Sex hormone receptors

CVMs can present in several anatomical sites besides the orbital region, such as the liver, uterus, breast, and brain. Lesions in these regions^(72-77)^ or in the orbital region^(24)^ may increase in size and trigger pain during pregnancy, in patients on hormone replacement therapy, or in those using oral contraceptives. Therefore, estrogen and progesterone levels may promote the growth of orbital CVMs. Furthermore, in postmenopausal women, when the hormone levels decrease, the lesions may stop growing or even regress.

Immunohistochemical assays have demonstrated the presence of progesterone receptors in the smooth muscle and endothelium of orbital CVMs; the presence of estrogen receptors has not yet been reported^(13,59)^. Although such evidence is strongly suggestive of a possible hormonal modulation in CVM progression, regression of the lesions without any medical intervention and in the absence of significant hormonal modulation has been reported in women^(78)^.

### Innate proliferative potential

The Ki-67 marker is a nuclear antigen associated with cell proliferation. Immunohistochemical studies of IHs have indicated positive staining for Ki-67 on endothelial cells; however, CVM specimens have demonstrated negative staining ^(11,61)^. This may be indicative of the low proliferative potential of CVMs, which supports the hypothesis that they are not true neoplasms. Their low proliferative potential can be evidenced even when they are incompletely excised^(34,79)^, with only anecdotal evidence of recurrence^(80,81)^. Smooth muscle cells of the injured vessels of CVMs demonstrate positive staining for ki-67, albeit to a lesser extent. This may indicate that the proliferation of these cells may be associated with CVM growth in addition to growth factors.

Table 1 summarizes the immunohistochemical profile of orbital CVMs and its possible implications.

**Table 1:** Immunophenotypic profile of the orbital cavernous venous malformation

Marker	Descrition	Specific cellular targets	Reativity	Possible implications
CD31	Platelet endothelial cell adhesion molecule-1 (PECAM-1); integral transmembrane protein.	Vascular endothelium	Positive	Formation of thrombosis and neovascular networks
CD34	Transmembrane sialomucin protein is expressed in early hematopoieticand vascular-associated tissues	Vascular and lymphatic endothelium	Positive	Formation of thrombosis and neovascular networks
D2-40	Podoplanin; O-linked sialoglycoprotein	Lymphatic endothelium	Negative	Absence of the lymphatic component
GLUT1	Glucose transporter type 1 protein	Microvascular endothelium of the barrier tissue	Negative	Positive markers for IHs
α-SMA	Smooth muscle actin	Smooth muscle cells, thin contractile filaments in vascular cells, pericytes, myofibroblasts, and myoepithelial cells	Positive	Presence of contractile myofilaments
Desmin	Type III intermediate anchoring filament in the muscle cells	Smooth and striated muscle and myocardial cells	Negative	Incomplete smooth muscle cell differentiation
ki-67	Nuclear proteins associated with cellular division	Nuclei of proliferating cells	Weakly positive	Low mitotic activity, proliferative phase of thrombus formation
PR	Progesterone receptors	Membranes of smooth muscle cells and endothelial cells	Positive	Possible hormonal modulation
VEGF	Vascular endothelial growth factor	Membrane of smooth muscle cells and endothelial cells	Positive	Neovascular remodulation and growth of the lesion

### Absence of an identifiable precursor and pathogenesis of the lesion

Vascular anomalies, despite being an important pathology and having a significant incidence rate, their origins are not yet established. Studies suggest that the pathogenesis of IHs is the clonal expansion of endothelial progenitor cells exposed to factors that favor their rapid proliferation, such as inadequate signaling or somatic mutations^(82)^. Several theories have been proposed regarding the identity of these progenitor cells. However, currently, it is believed that hemangioma-derived stem cells (Hem-SCs), a multipotent progenitor normally present during embryonic development, can persist in an immature state of development. The dysregulation of Hem-SCs orchestrates the pathophysiology of IH through various regulatory and signaling pathways^(43,83)^.

There is no histological evidence of a single rapidly proliferating precursor from which CVMs could originate ^(43)^. Some theories include the possibility of orbital CVMs arising from a preexisting collapsed congenital lesion, which may have originated from an unidentified multipotent endothelial precursor^(11)^.

Regardless of the diversity in the clinical phenotypes of vascular malformations, several common genes and signaling pathways are involved in their pathogenesis. The observed morphogenetic defects may be caused by a genetic factor, perinatal adverse event, or a postnatal secondary hit or trigger on a preexisting genetic defect^(4,84,85)^. The phenotypic variability of vascular malformations can be explained by several factors that can influence the final expression of a genetic mutation, such as somatic mosaicism^(4)^, mutations during embryonic development, mutation type, frequency and penetrance of the mutant allele, and hemodynamic forces that can gradually and permanently reprogram endothelial cells, radically altering their morphology and function^(85-87)^.

Most vascular malformations are caused by mutations in genes involved in three signaling pathways: (a) PIK3CA/mTOR pathway, in which mutations could lead to a malformation with associated overgrowth; (b) RAS/MAPK pathway, which is mainly involved in fast-flowing arteriovenous malformations; and (c) G protein-coupled receptor signaling, which is involved in capillary malformations and congenital hemangiomas^(88-93)^.

Among vascular malformations, involvement of genetic changes in their pathogenesis was first identified in venous malformations. In 1996, the presence of mutations in the TEK gene in blood samples from patients with familial mucocutaneous venous malformations was identified^(93)^, which was also seen in subsequent studies of other types of venous malformations^(94-100)^.

In the periorbital region, mutations are observed in genes that play an important role in pathways involved in angiogenesis, vascular cell growth, apoptosis, and proliferation, mainly in the RAS/mitogen-activated protein kinase pathway/extracellular signal-regulated kinase (RAS/Raf/MEK) and the p53/mTOR pathway. Mutations in genes that are possibly involved in these pathways, such as MC4R, c-Kit, and GJA4, have been found in cavernous lesions in the orbital region^(94,99-100)^. Table 2 summarizes the main genetic mutations related to cavernous vascular lesions.

**Table 2:** Genetic mutations in orbital cavernous venous malformations

Site	Descrition	Biological association	Possible implications
MC4R	G protein-coupled melanocortin receptor	Cell cycle, proliferation, and migration of endothelial cells	Increased angiogenic activity of endothelial cells
C-kit	Receptor tyrosine kinase encoded by a proto-oncogene.	Proliferation, differentiation, adhesion, survival, and programed cell death	Aberrant cellular behaviors in the vessel walls
GJA4	Isoforms of connexin proteins	Angiogenesis, remodeling, and vascular permeability	Increased hemichannel activity and alteration of integrity and functionality of endothelial cells

Although these genes have been identified in several types of vascular lesions, including cavernous lesions of the orbit, the mechanisms that translate the information contained in them into different clinical spectrums remain unknown.

It is not implausible that orbital CVMs may appear only in adulthood as new lesions without the presence of any preexisting vascular defect.

There is evidence of the occurrence of postnatal angiogenesis and vasculogenesis in areas of hemodynamic stress or other injuries, which would lead to the activation of cellular progenitors (local or chemotactically recruited) that should remain quiescent under normal physiological conditions^(43)^. Furthermore, not all congenital lesions may be present at birth. Depending on their size or growth rate, some congenital vascular lesions may not be identifiable until adulthood, as with CVMs^(46,47)^.

### Atypical malformation

Although the compelling evidence about their biological status points to a malformation and not a tumor in the classical sense of the word, orbital CVMs do not perfectly fit the definition of malformations^(43)^. For example, orbital CVMs share histological characteristics with CVMs of other anatomical regions (mainly skin and liver), such as the presence of dilated blood vessels lined by flattened endothelial cells. However, orbital CVM differs from these other malformations in the degree of encapsulation of the lesion, occurrence of intralesional thrombosis, and stromal expansion.

Furthermore, the characteristics of the clinical course of the disease and its development and growth patterns may indicate that CVMs are an atypical example of malformation.

The use of a classification for vascular lesions that change over time and that has never reached a consensus is potentially problematic because it limits a multidisciplinary approach and hinders the development of new therapeutic modalities. A robust and unambiguous categorization is required for vascular anomalies, especially those of the orbit.

The current set of clinical, morphological, and biological evidence suggests that despite some confusion in historical nomenclatures and their peculiarities, the so-called cavernous hemangiomas of the orbit are actually CVMs.
